# Integrity of lipid nanocarriers in bloodstream and tumor quantified by near-infrared ratiometric FRET imaging in living mice

**DOI:** 10.1016/j.jconrel.2016.06.027

**Published:** 2016-08-28

**Authors:** Redouane Bouchaala, Luc Mercier, Bohdan Andreiuk, Yves Mély, Thierry Vandamme, Nicolas Anton, Jacky G. Goetz, Andrey S. Klymchenko

**Affiliations:** a Laboratoire de Biophotonique et Pharmacologie, UMR CNRS 7213, University of Strasbourg, 74 route du Rhin, 67401 Illkirch Cedex, France; b Laboratory of Photonic Systems and Nonlinear Optics, Institute of Optics and Fine Mechanics, University of Setif 1, 19000, Algeria; c MN3T, Inserm U1109, LabEx Medalis, Fédération de Médecine Translationnelle de Strasbourg (FMTS), University of Strasbourg, F-67200, France; d Organic Chemistry Department, Chemistry Faculty, Taras Shevchenko National University of Kyiv, 01601 Kyiv, Ukraine; e CNRS UMR 7199, Laboratoire de Conception et Application de Molécules Bioactives, University of Strasbourg, 74 route du Rhin, 67401 Illkirch Cedex, France

**Keywords:** Lipid nanocarriers, *In vivo* imaging, Near-infrared FRET, Nanocarrier integrity, Enhanced permeability and retention, Tumor

## Abstract

Lipid nanocarriers are considered as promising candidates for drug delivery and cancer targeting because of their low toxicity, biodegradability and capacity to encapsulate drugs and/or contrasting agents. However, their biomedical applications are currently limited because of a poor understanding of their integrity *in vivo*. To address this problem, we report on fluorescent nano-emulsion droplets of 100 nm size encapsulating lipophilic near-infrared cyanine 5.5 and 7.5 dyes with a help of bulky hydrophobic counterion tetraphenylborate. Excellent brightness and efficient Förster Resonance Energy Transfer (FRET) inside lipid NCs enabled for the first time quantitative fluorescence ratiometric imaging of NCs integrity directly in the blood circulation, liver and tumor xenografts of living mice using a whole-animal imaging set-up. This unique methodology revealed that the integrity of our FRET NCs in the blood circulation of healthy mice is preserved at 93% at 6 h of post-administration, while it drops to 66% in the liver (half-life is 8.2 h). Moreover, these NCs show fast and efficient accumulation in tumors, where they enter in nearly intact form (77% integrity at 2 h) before losing their integrity to 40% at 6 h (half-life is 4.4 h). Thus, we propose a simple and robust methodology based on ratiometric FRET imaging *in vivo* to evaluate quantitatively nanocarrier integrity in small animals. We also demonstrate that nano-emulsion droplets are remarkably stable nano-objects that remain nearly intact in the blood circulation and release their content mainly after entering tumors.

## Graphical abstract

**Figure:**
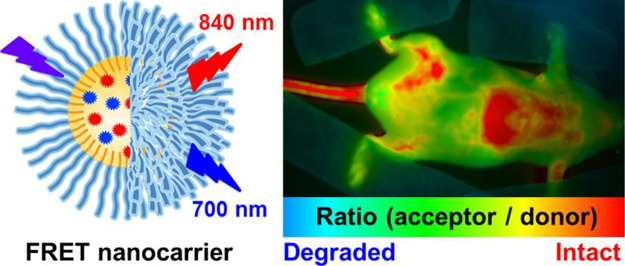

## 1 Introduction

**Fig. 1:**
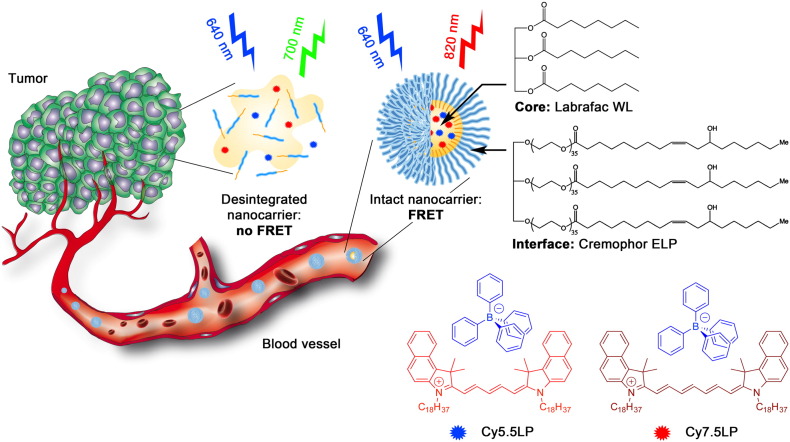
Concept of FRET NCs that can report on their integrity by change in their emission color. Chemical structures of oil Labrafac WL (medium chain triglyceride) and Cremophor ELP (PEGylated surfactant) as well as lipophilic cyanine 5.5 and 7.5 dyes (Cy5.5LP and Cy7.5LP) with their bulky hydrophobic counterions are shown.

Nanoscale vehicles (nanocarriers, NCs) become indispensable tools for *in vivo* imaging [1], [2], [3], drug delivery [4], [5] and image-guided surgery [6], [7]. One key requirement that any NC has to meet is to maintain its integrity until it reaches the target, for example a tumor [8], [9]. This would ensure robust delivery of active molecules and/or provide the best signal to noise ratio when NCs are used as contrasting agents. However, suitable methods for assessing nanocarrier stability and the cargo leakage (such as dialysis, size exclusion chromatography, FCS, etc) are sparse and mostly operate *in vitro* [10]. Therefore, while providing useful information, these model experiments cannot resolve fundamental issues such as the passage of NCs through the bloodstream, which includes shear forces, opsonization and uptake, and other undesirable interactions with off-target cells [11], [12], [13]. Moreover, in the context of tumor targeting, it is of utmost importance to assess whether NCs are capable of maintaining their load upon extravasation from the systemic circulation into the tumor (Fig. 1). Additionally, a reliable method for assessing their integrity can inform on the time scale of release of NCs' content upon tumor targeting through EPR (enhanced permeation and retention) effect [14], [15]. Therefore, it is essential to monitor the integrity of the nanocarriers *in vivo*, preferably in real time. Current methods to study the integrity of the nanocarriers *in vivo* are very limited. In addition to radiolabelling assays [16], the highly promising approach is fluorescence imaging in the near-infrared (NIR) region, which now ranges from classical 2D imaging of small animals up to fluorescence-mediated tomography that enables quantitative 3D imaging [17], [18], [19], [20]. Importantly, optical imaging modality provides access to Förster Resonance Energy Transfer (FRET), which acts as a molecular ruler between the donor and acceptor dyes and has been extensively used to characterize properties of bio-/nano-materials and their response to biological environments [13], [21], [22], [23]. It is particularly suitable to study the integrity of a nanocarrier, because of exquisite sensitivity of FRET to changes in the donor acceptor distance. Thus, encapsulation of both donor and acceptor inside a nanocarrier should ensure high FRET efficiency, while the loss of the nanocarrier integrity associated with the release of its components into the medium should result in the loss of the FRET signal. Moreover, when FRET occurs between two fluorescent dyes, dual emission of NCs can be obtained, which opens possibilities for quantitative ratiometric measurements using fluorescence detection in two distinct optical windows. It is only very recently that FRET imaging has been used for monitoring *in vivo* the integrity of NCs. It has been successfully applied to monitor biodistribution and integrity of polymer micelles [24], hybrid organic-inorganic nanoparticles [9] and nanoemulsion droplets [25]. However, these studies only showed global FRET signal from the mice or a tumor, without imaging directly the particle integrity in the blood circulation, where these nanocarriers are actually injected. For this purpose, only indirect *ex vivo* measurements and intravital microscopy, which requires complex dedicated setup, were realized to date [9], [26], [27]. Moreover, the studies were limited to qualitative evaluation and no quantification of nanocarrier integrity directly in living mice was reported to date. Quantitative *in vivo* fluorescence imaging of NCs integrity can be achieved only after some key limitations of existing fluorescent NCs are addressed. The first limitation is the insufficient fluorescence signal from NCs that is contaminated by the background from animal tissue. This could be overcome by high dye loading into NCs with minimal self-quenching of the encapsulated dyes and injecting large quantities of NCs into mice without toxic effects. Moreover, the loaded dyes should operate in the NIR region (> 700 nm), where the absorption, light-scattering and auto-fluorescence of the tissue are minimal [28], [29], [30]. Second, intensity of the FRET signal was usually analyzed, making the assessment of nanocarrier integrity only qualitative. Quantification requires internal control, which can be realized by ratiometric FRET imaging, obtained by division of two images (*e.g.* acceptor/donor). In contrast to intensity, the measured ratio values are absolute, being independent of concentration of emissive species and light source intensity [31], [32]. However, only one rare example used ratio imaging in application to analyze FRET of nanocarriers *in vivo* [33]. The problem is that in majority of examples FRET donor emits below near-infrared (NIR) window (< 700 nm), so that its signal is strongly attenuated by tissue absorption and contaminated by light scattering and auto-fluorescence. Therefore, quantitative and reliable ratiometric *in vivo* imaging requires both donor and acceptor species to emit in the near-infrared (NIR) region above 700 nm.

Among the existing nanocarriers for drugs and contrasting agents in the biomedical research, lipid nano-emulsion emerged recently as a promising alternative [34], [35], [36]. Although nano-emulsions were mainly used for the last > 20 years as template for nanoparticle preparation [37], only in the last years they attracted attention as nano-carriers in pharmacy and cosmetics application [38], [39]. Nano-emulsifications enable preparation of lipid nanocarriers of well-defined size from FDA approved materials using direct approaches that do not use organic solvents [40]. This feature is particularly attractive for high quality fluorescence imaging as it enables injecting maximal amount of nanocarriers with minimal harm to an animal [34], [35]. However, as nano-emulsions are essentially liquid objects, two major problems need to be addressed before they can be broadly used in drug delivery and/or as contrasting agents. First, efficient encapsulation of material and active compounds, without fast leakage, needs to be achieved. Second, *in vivo* stability is required to ensure proper delivery. Both issues are actively debated over the last years [25], [41], [42], [43]. Recently, we introduced an original concept to improve the loading of cyanine dyes into the oil droplet, which is based on the use of hydrophobic counterions [44]. Cyanine dyes usually bear inorganic counterions such as perchlorate or iodide that are responsible for poor solubility of these dyes in oils. By replacing these ions with tetraphenyl borate (TPB), we were able to improve solubility > 40-fold and create 90 nm lipid droplets bearing exceptional number of cyanine dyes (~ 12,000 per droplet, 8 wt% dye loading) that remained efficiently fluorescent [44]. The extreme brightness of these NCs enabled pioneer single particle tracking *in vivo* in a zebrafish embryo model. However, this unique counterion approach has never been applied to NIR cyanine dyes, required for *in vivo* imaging, and it has never been used to generate FRET inside nanocarriers, needed to assess the NCs integrity.

In the present work, we synthesized lipophilic cyanine 5.5 and 7.5 dyes bearing long alkyl chains and TPB counterion (Cy5.5LP and Cy7.5LP, respectively, Fig. 1) and encapsulated them inside lipid nanocarriers at concentrations required for highly efficient FRET, *i.e.* 1% in oil. These dyes ideally fit the NIR spectral region for *in vivo* imaging, where, notably, our cyanine 7.5 is an apolar analogue of indocyanine green commonly used for *in vivo* imaging [45], [46]. The obtained ultrabright FRET NCs were successfully injected and monitored in healthy and tumor-bearing mice. We were able, for the first time, to visualize and quantitatively assess lipid nanocarrier integrity directly in the bloodstream, liver and tumor of living mice. We showed that lipid NCs remain nearly intact in the blood circulation, and they can enter the tumor with minimal loss of their integrity followed by the release their content with a half-life of 4.4 ± 0.3 h. Thus, we provide a robust imaging methodology to follow the evolution of lipid NCs *in vivo*, showing their remarkable stability *in vivo* and capacity to deliver their content into the target cancer tissues.

## 2 Materials and methods

### 2.1 Materials

All chemicals and solvents for synthesis were from Sigma Aldrich. Cremophor ELP® (Kolliphor ELP®) was provided by BASF (Ludwigshafen, Germany), Labrafac WL® (medium chain triglycerides) by Gattefossé (Saint-Priest, France). Ultrapure water was obtained using a MilliQ® filtration system (Millipore, Saint-Quentin-en-Yvelines, France). Fetal bovine serum (FBS) was acquired from Lonza (Verviers, Belgium) and Gibco-Invitrogen (Grand Island, USA).

### 2.2 Synthesis of Cy5.5LP and Cy7.5LP

#### 2.2.1 1,1,2-trimethyl-3-octadecyl-1H-benzo[*e*]indol-3-ium iodide (**1**)

250 mL round-bottom flask equipped with magnetic stirring bar was charged with 1,1,2-trimethylbenz[*e*]indole (1 eq., 6.88 g, 32.9 mmol) and 1-iodooctadecane (2 eq., 25 g, 65.7 mmol), 100 mL of 2-butanone was added subsequently. Reaction mixture was refluxed for 24 h, then cooled down to r.t. Reaction mixture was cooled down to r.t., diethyl ether was added and formed solid part was filtered off and washed with 100 mL of diethyl ether. Obtained crystals of crude product were redissolved in DCM and precipitated back while by adding diethyl ether, afterwards filtered and washed with diethyl ether. Product was obtained as slightly green crystals in 76% yield (14.73 g).

^1^H NMR (400 MHz, CDCl_3_) *δ* 8.10 (d, *J* = 8.7 Hz, 1H), 8.08 (dd, *J* = 8.0, 1.1 Hz, 1H), 8.04 (dd, *J* = 8.2, 1.3 Hz, 1H), 7.76 (d, *J* = 8.9 Hz, 1H), 7.72 (ddd, *J* = 8.3, 6.9, 1.4 Hz, 1H), 7.65 (ddd, *J* = 8.1, 6.9, 1.2 Hz, 1H), 4.78 (t, *J* = 7.7 Hz, 2H), 3.19 (s, 3H), 1.97 (p, *J* = 7.8 Hz, 2H), 1.87 (s, 6H), 1.52–1.41 (m, 2H), 1.40–1.30 (m, 2H), 1.28–1.19 (m, 26H), 0.85 (t, *J* = 7.0 Hz, 3H).

^13^C NMR (100 MHz, CDCl_3_) *δ* 195.25, 138.34, 137.29, 133.82, 131.56, 130.17, 128.77, 127.97, 127.74, 122.96, 112.59, 56.04, 50.56, 32.00, 29.77, 29.76, 29.73, 29.70, 29.65, 29.55, 29.43, 29.41, 29.24, 28.26, 26.92, 22.85, 22.76, 17.03, 14.18.

HRMS (*m*/*z*): [M]^+^ calcd for C_33_H_52_N 462.40943; found 462.40854.

#### 2.2.2 Dioctadecylcyanine 5.5 chloride (**2**)

1,1,2-trimethyl-3-octadecyl-1H-benzo[*e*]indol-3-ium iodide (**1**) (1 eq., 2 g, 3.39 mmol) was placed in 50 mL round-bottom flask. 10 mL of dry pyridine was added *via* syringe. Then, 1,1,3,3-tetramethoxypropane (1.5 eq., 0.835 g, 0.838 mL, 5.09 mmol) was quickly added dropwise to the boiling solution of indoleninium salt using syringe. Reaction mixture was stirred under reflux for 3 h. After cooling down to room temperature solvent was removed under reduced pressure. To the obtained residue 50 mL of dichloromethane were added. Obtained solution was washed three times with 1 N HCl, then with brine and water. The crude product was purified by flash column chromatography on silica gel using ethyl acetate/dichloromethane (9:1) mixture as eluent. Cyanine was obtained as dark blue-greenish viscous oil in 76% yield (2.8 g).

^1^H NMR (400 MHz, CDCl_3_) *δ* 8.55 (t, *J* = 13.0 Hz, 2H), 8.20 (d, *J* = 8.5 Hz, 2H), 7.90 (d, *J* = 8.6 Hz, 4H), 7.64–7.54 (m, 2H), 7.44 (t, *J* = 7.5 Hz, 2H), 7.32 (d, *J* = 8.8 Hz, 2H), 6.74 (t, *J* = 12.4 Hz, 1H), 6.24 (d, *J* = 13.7 Hz, 2H), 4.14 (t, *J* = 7.5 Hz, 4H), 2.12 (s, 12H), 1.83 (p, *J* = 7.6 Hz, 4H), 1.46 (p, *J* = 7.6, 7.0 Hz, 4H), 1.40–1.33 (m, 4H), 1.30–1.18 (m, 52H), 0.85 (t, *J* = 6.7 Hz, 6H).

^13^C NMR (100 MHz, CDCl_3_) *δ* 174.60, 153.33, 139.40, 134.46, 131.81, 130.44, 129.94, 128.32, 127.83, 125.11, 122.65, 110.44, 103.10, 51.53, 44.64, 32.00, 29.79, 29.77, 29.74, 29.70, 29.66, 29.55, 29.48, 29.44, 27.93, 27.80, 27.08, 22.77, 14.21.

HRMS (*m*/*z*): [M]^+^ calcd for C_69_H_103_N_2_ 959.81158; found 959.8098.

#### 2.2.3 Dioctadecylcyanine 7.5 chloride (**3**)

1,1,2-trimethyl-3-octadecyl-1H-benzo[*e*]indol-3-ium iodide (**1**) (2.2 eq., 1029 mg, 1.75 mmol) and glutaconaldehydedianil hydrochloride (1 eq., 226 mg, 0.794 mmol) were mixed in 10 mL of pyridine, afterwards Ac_2_O (13.4 eq., 1087 mg, 1 mL, 10.6 mmol) was added and the reaction mixture was heated to 60 °C while stirring and left for 3 h. After reaction was finished, solvents were evaporated at vacuum, and the crude product was dissolved in DCM, washed with 0.1 N HCl (3 times), brine and water. DCM layer was dried over Na_2_SO_4_, the solvent was evaporated and the product was purified by column chromatography on silica (gradient DCM/MeOH 99/1–95/5). Product was obtained as a green solid (926 mg, 0.906 mmol, 52%).

^1^H NMR (400 MHz, CDCl_3_): *δ* 8.16 (d, *J* = 8 Hz, 2H), 8.06 (t, *J* = 12 Hz, 2H), 7.97 (bs, 1H), 7.92 (d, *J* = 8 Hz, 4H), 7.61 (t, *J* = 7 Hz, 2H), 7.46 (t, *J* = 7 Hz, 2H), 7.35 (d, *J* = 9 Hz, 2H), 6.67 (t, *J* = 12 Hz, 2H), 6.25 (d, *J* = 12 Hz, 2H), 4.14 (bs, 4H), 2.04 (s, 12H), 1.87 (m, *J* = 7 Hz, 4H), 1.49 (m, *J* = 7 Hz, 4H), 1.39 (m, *J* = 7 Hz, 4H), 1.26 (bs, 52H), 0.88 (t, *J* = 7 Hz, 6H).

^13^C NMR (100 MHz, CDCl_3_): *δ* 173.07, 157.04, 151.02, 139.74, 133.98, 131.83, 130.59, 130.09, 128.41, 127.87, 126.27, 125.06, 122.47, 110.56, 103.39, 51.155, 44.76, 32.06, 29.842, 29.807, 29.76, 29.72, 29.60, 29.53, 29.50, 27.86, 27.14, 22.82, 14.26.

HRMS (*m*/*z*): [M]^+^ calcd. For C_71_H_105_N_2_^+^ 985.8272; found 985.8290.

#### 2.2.4 Dioctadecylcyanine 5.5 tetraphenylborate (Cy5.5LP)

Dioctadecylcyanine 5.5 chloride (1 eq., 100 mg, 0.1 mmol) was dissolved in 5 mL of DCM, sodium tetraphenylborate (3 eq., 103 mg, 0.301 mmol) was added and the dispersion was sonicated for 5 min. TLC control has shown full conversion. Afterwards, the mixture was purified on a silica column, eluent DCM/MeOH 95/5 (product goes almost with front). Dioctadecylcyanine 5.5 tetraphenylborate (109.2 mg, 0.085 mmol, 85%) was obtained as blue-green viscous oil and used without further characterisation.

#### 2.2.5 Dioctadecylcyanine 7.5 tetraphenylborate (Cy7.5LP)

Dioctadecylcyanine 7.5 chloride (1 eq., 200 mg, 0.18 mmol) was dissolved in 5 mL of DCM, sodium tetraphenylborate (3 eq., 184 mg, 0.539 mmol) was added and the dispersion was sonicated for 5 min. TLC control has shown full conversion. Afterwards, the mixture was purified on a silica column, eluent DCM/MeOH 95/5 (product goes almost with front). Dioctadecylcyanine 7.5 tetraphenylborate (218 mg, 0.167 mmol, 93%) was obtained as green viscous oil and used without further characterisation.

### 2.3 Formulation and characterisation of lipid nanocarriers

Dye loaded nanoemulsions were produced by spontaneous nano-emulsification. Briefly, the dyes (Cy5.5LP and Cy7.5LP) were firstly dissolved in Labrafac WL® (56 mg) at concentrations ranging from 0.1 to 1% by weight. Then, Cremophor ELP® (also called Kolliphor ELP®) was added (44 mg), and the mixture was homogenized under magnetic stirring at 37 °C for 10 min up to complete homogenisation. Finally, nanoemulsions were generated with the addition of ultrapure (Milli-Q) water (230 mg). Size distributions were determined by dynamic light scattering using a Zetasizer Nano series DTS 1060 (Malvern Instruments S.A).

### 2.4 Fluorescence spectroscopy

Absorption and fluorescence spectra were recorded on a Cary 4 spectrophotometer (Varian) and a Fluoromax 4 (Jobin Yvon, Horiba) spectrofluorometer, respectively. Fluorescence emission spectra were performed at room temperature with 670 and 760 nm excitation wavelengths for Cy5.5LP and Cy7.5LP loaded nanocarrier, respectively. The emission spectra were corrected from for the wavelength-dependent response of the detector. All fluorescence measurements were done using solutions with absorbance ≤ 0.1 at the wavelength of excitation. The relative fluorescence quantum yield was measured using DiD dye in methanol (QY = 33%) as reference using excitation at 630 nm [47].

### 2.5 FRET-based stability test

The stability of lipid nanocarriers was estimated using Forster resonance Energy transfer (FRET) between two encapsulated dyes, 1% of Cy5.5LP (with respect to Labrafac WL®) as energy donor and 1% of Cy7.5LP as energy acceptor were used. The NCs were diluted 10,000 times from the original formulation and incubated in water and 100% of fetal bovine serum (FBS). High dilution was needed to avoid saturation of serum by lipids of NCs. The donor in the nanocarriers was excited at 670 nm. The semi-quantitative parameter of FRET efficiency was calculated according to the following equation E = A/(A + D) [48], where A and D are the maximum of fluorescence intensity of the acceptor and donor, respectively.

### 2.6 Cytotoxicity studies

In 96-well plates, HeLa cells were seeded at a concentration of 1 × 10^4^ cells per well in 200 μL of the DMEM growth medium and then incubated overnight at 37 °C in humidified atmosphere containing 5% CO_2_. Next, we add the lipid Nanocarriers (1% Cy5.5LP–Cy7.5LP), by substituting the culture medium for a similar one containing variable dilutions of the Nanocarriers. After incubation for 24 h, the medium was removed. Then, the wells were filled with cell culture medium containing MTT, incubated for 4 h at 37 °C, and the formazan crystals formed were dissolved by adding 100 μL of DMSO and shaked for 10 min. We measure the absorbance at 570 nm with a microplate reader (Xenius, Safas). Experiments were carried out in triplicate, and expressed as a percentage of viable cells compared to the control group.

### 2.7 Subcutaneous tumor grafting and administration of FRET nanocarriers

Adult (10 months) immuno-deficient mice (NMRI-Foxn1nu/Fox1nu, Janvier labs, 1) were anesthetized *via* gas anesthesia (isoflurane) system prior to tumor cell grafting. Anesthetized mice were injected subcutaneously (in the flank) with 100 μL of a solution made of 50% PBS and 50% Matrigel containing 1.10^6^ of D2A1 (murine mammary carcinoma) cells [49]. Tumors were grown over a period of 20 days before administrating lipid nanocarriers. The mouse studies were performed according to the Guide for Care and Use of Laboratory Animals (E67-6-482-21) and the European Directive with approval of the regional ethical committee (CREMEAS for Comité Régional d'Ethique en Matière d'Expérimentation Animale de Strasbourg, AL/73/80/02/13). Mice received food and water *ad libitum*; they were checked daily and tumor growth never exceeded 20 days, leading to low-size tumors with no impact on the animal's health. All efforts were made to minimize suffering and euthanasia was performed using CO2. General health status was monitored regularly by independent observers. Sacrifice of the animal was effectuated when reaching limit ethical endpoints. Before administration of the nanocarriers solution, mice were anesthetized by intraperitoneal injection of a mixture of ketamine (100 mg/kg) and xylazine (10 mg/kg). The nanocarriers were administrated by *retro*-orbital injection (100 μL) as previously performed for other purposes [50].

### 2.8 *In vivo* whole animal FRET imaging

Whole- and live-animal imaging of the FRET signal in healthy and tumor-bearing mice was performed by using a luminograph (NightOwl, Berthold Technologies). Anesthetized mice (isoflurane) were placed repeatedly in the luminograph, and positioned either on the flank or the back. Mice were imaged using a halogen lamp, (75 W, 340–750 nm) and emission of the two dyes was collected separately using separate filters sets (630/700 nm for Cy5.5LP, and 630/820 nm for Cy7.5LP). The experiments with healthy and tumor bearing mice were repeated three times.

### 2.9 Calibration of the ratiometric response of NCs to disintegration

To calibrate the ratiometric response of NCs to disintegration in our *in vivo* imaging setup, we model the disintegration NCs by mixing intact FRET NCs with NCs containing separately donor and acceptor at low concentration, with the preservation of the same concentration of dyes: 100% integrity corresponds to FRET NCs (1% of Cy5.5LP and 1% of Cy7.5LP) diluted in PBS 1000-fold from the original formulation, while 0% integrity corresponds to a mixture of NCs containing separately donor (0.1% of Cy5.5LP) and acceptor (0.1% Cy7.5LP), both diluted at 100-fold in PBS. Intermediate mixtures were made, where the level of integrity (%) is defined as the fraction of Cy5.5LP/Cy7.5LP dyes in the FRET NCs with respect to the total amount of these dyes. They were placed into 96-well plate and imaged using the NightOwl setup. The measurements were done in triplicate. The obtained values of the A/(A + D) ratio were plotted *vs* % of integrity and the data were fit with an exponential function (1):

(1) $$y=a+be^{cx}$$

where *a* = 0.14572; *b* = 0.1254; *c* = 0.01748, *y* is A/(A + D) and *x* is FRET pair concentration (wt%).

As another model of the complete disintegration of NCs, solution FRET NCs diluted 1000-fold in dioxane was placed into 1 mL Eppendorf® tube and imaged by the NightOwl setup.

### 2.10 Image analysis of the FRET signal in the living mice

The ratiometric images were built using a homemade plugin (developed by Romain Vauchelles) under ImageJ that divides the image of the 840 nm channel by that of the 700 nm channel. For each pixel, a pseudocolor scale is used for coding the ratio, while the intensity is defined by the integral intensity recorded for both channels at the corresponding pixel. Image analysis was performed by using the ImageJ software [51]. The tail vein, liver and tumor were manually delimitated and the mean intensity of the delimitated region was determined for each respective time point and channel for three healthy and three tumor-bearing mice. For both donor and acceptor channels, the signal was corrected for the background using the mice before injection (in the corresponding region of interest). Then the corrected values of A/(A + D) were converted into the FRET pair concentration using Eq. (1). The obtained values of FRET pair concentration were then plotted *vs* time and fitted using a logistic function (2):

(2) $$y=a_{2}+\frac{\left(a_{1}-a_{2}\right)}{(1+{\left(\frac{x}{x_{0}}\right)}^{p}}$$

where *y* is the FRET pair concentration (wt%); *x* is time; other parameters were calculated during the fitting procedure. This fit was further used to calculate the integrity half-life.

### 2.11 Statistical analysis

Student's *t*-test was used to evaluate the statistical significance between two sample groups. The differences between the results were considered to be significant when the *p*-values were < 0.05.

## 3 Results and discussion

**Fig. 2:**
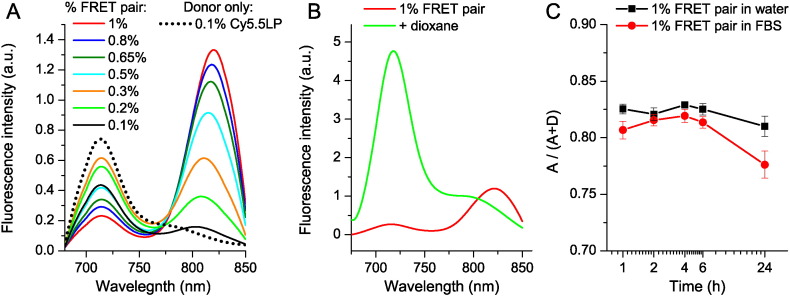
FRET in the lipid NCs. (A) Fluorescence spectra of dye-loaded NCs as a function of weight % of FRET pair (corresponds to % of each dye). The spectra were recorded for the same 1000-fold dilution of NCs in water. (B) Disintegration of NCs by dioxane: fluorescence spectra of NCs encapsulating 1% of Cy5.5LP and 1% of Cy7.5LP diluted 1000-fold in water or in dioxane. (C) Stability of FRET NCs in serum: FRET signal, expressed as A/(A + D) ratio, for different incubation times in 100% serum or water at 37 °C. To avoid saturation of serum by lipids of NCs, 10,000-fold dilution from original formulation was used. Excitation wavelength was systematically 670 nm.

Nanoemulsions were selected as NCs for several reasons. First, they are very efficient at encapsulating materials. Second, nanoemulsions can be easily formulated by spontaneous emulsification with non-toxic compounds compatible with the parenteral administration route, so that they can be intravenously injected at high concentrations into mice with minimal harm [34], [35]. The latter is particularly attractive to achieve superior contrast for imaging nanocarriers directly in the blood circulation. Cyanines 5.5 and 7.5 dyes were selected for encapsulation, because they are an excellent FRET couple with ideal spectral properties for *in vivo* whole-animal imaging. Indeed, cyanine 5.5 derivatives are among the most commonly used NIR imaging agents with convenient excitation 650–700 nm and emission in NIR region (> 700 nm). Moreover, cyanine 7.5 absorption spectrum overlaps perfectly with the emission spectrum of cyanine 5.5 (Fig. S2), while its emission in NIR region at 840 nm is perfectly separated from the emission of cyanine 5.5, making this couple perfectly tailored for ratiometric FRET imaging. To achieve both strong brightness and highly efficient FRET inside NCs, cyanines 5.5 and 7.5 should be encapsulated at very high concentrations up to 1 wt% in oil, as we earlier showed for other FRET pair [41]. Therefore, their hydrophobic analogues, dioctadecylcyanines 5.5 (**2**) and 7.5 (**3**), were synthesized bearing long alkyl chains to ensure their high lipophilicity. Nevertheless, their solubility in labrafac oil (medium chain triglycerides) was only 0.15 and 0.05 wt%, for **2** and **3**, respectively. Therefore, we replaced their inorganic counterion (chloride) with bulky hydrophobic tetraphenylborate, which, according to our earlier study, improved drastically the solubility of the cyanine 3 dye DiI [44]. The obtained new salts, Cy5.5LP and Cy7.5LP (Fig. 1), showed much higher solubility in labrafac, 7 and 4 wt%, respectively. Then, nano-emulsions (lipid NCs) were generated by the spontaneous emulsification of dye-loaded oil and non-ionic surfactant (Cremophor® ELP), giving rise to fluorescent PEGylated droplets sizing around 90–100 nm (Table S1). To generate FRET NCs, we prepared NCs encapsulating increasing amount of Cy5.5LP and Cy7.5LP. As expected, the emission spectra recorded after excitation of the energy donor showed strong dependence on the concentration of the FRET pair (Fig. 2A). Thus, at 0.1% of dyes, the emission spectrum was very close to that of Cy5.5LP alone, while at higher concentrations, a long wavelength emission, corresponding to the FRET acceptor, appeared and became dominant at ≥ 0.5% of the dyes. It should be noted that the emission maximum of Cy7.5LP shifted gradually to the red with increase in the dye content (Fig. 2A). Similar concentration-dependent red shifts were previously observed for Cy3 dye in lipid droplets [44]; they could be related to some aggregation of the dyes, homo-FRET, as well as to small changes in the oil core properties at higher dye concentrations. Remarkably, the total (donor + acceptor) fluorescence quantum yield (QY) of NCs encapsulating FRET pair at 1% loading of each dye was 11%. Although this value was lower than the QY of NCs with 1% of Cy5.5LP alone (27%), it remained relatively high to ensure high brightness to these NCs, important for *in vivo* imaging. For comparison, QY of NIR dye indocyanine green measured in phosphate-buffered saline (PBS) is 2.4% [52]. Here, we achieved much higher QY already for the FRET system. Moreover, because NCs minimize potential interactions of loaded dyes with tissues *in vivo*, they allow the use of higher dye concentrations, which is essential for achieving optimal imaging contrast for NIR imaging.

Then, to verify the response of our FRET NCs to their disintegration, we forced this process by adding excess of water miscible organic solvent dioxane, which should destroy NCs by solubilizing its components. As a result, a strong emission of Cy5.5LP dye was observed, while the emission of Cy7.5LP was fully hampered (Fig. 2B). This result confirmed that integrity of NCs, where Cy5.5LP and Cy7.5LP are in very close proximity within the NCs, can be assessed based on FRET signal. We next assessed the stability of these FRET NCs by incubating them in serum (100%), used as model of a biological medium *in vivo*. As a semi-quantitative parameter of FRET efficiency, we used the band intensity ratio of acceptor to the sum of acceptor and donor, A/(A + D), so-called proximity ratio [48], [53]. This ratio showed only a mild decrease over 24 h of incubation in serum (Fig. 2C) and nearly no change in water, demonstrating the relative stability of our FRET NCs. Therefore, we concluded that these new FRET NCs, owing to good stability and relative ease in monitoring their integrity, should be perfectly suited for whole-animal NIR imaging through intravenous injection.

Before testing the integrity of our NCs *in vivo*, we evaluated cytotoxic effects of NCs by performing a cell viability assay *in vitro* on tumor cells. In the dilution range between 10,000 and 500, blank and dye-loaded FRET NCs did not show any significant cytotoxicity (Fig. S3). NCs were however cytotoxic when used at a 200-fold (and below) dilution. Importantly, the presence of both dyes (Cy5.5LP and Cy7.5LP) in the NCs had no added cytotoxic effect. It should be noted that the dilution factor that we further used in our mice experiments corresponded to ~ 1000-fold, which is well in the low cytotoxicity range according to the present data.

**Fig. 3:**
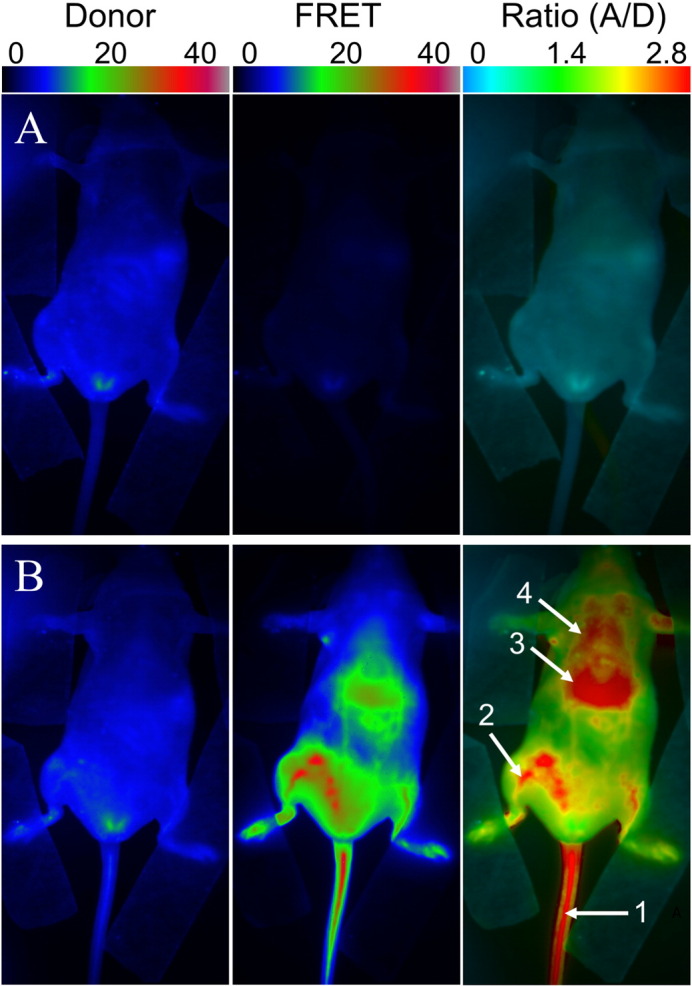
FRET imaging of healthy nude mice before (A) and after (B) injection of NIR-FRET lipid NCs (1% of Cy5.5LP and Cy7.5LP each). Left panels present intensity images of the Cy5.5LP channel (700 nm), middle panels present images of Cy7.5LP channel (820 nm), while the right panels present ratiometric images (acceptor/donor). The excitation wavelength was 630 nm. Numbers of arrows show vasculature of tail (1) and back leg (2) as well as liver (3) and lungs (4).

We then performed whole-animal *in vivo* NIR imaging of anesthetized and immobilized nude mice (Fig. S4) upon parenteral venous administration (retro-orbital) of the FRET NCs suspension, diluted to 50-fold in PBS (the dye concentration in the injected solution was 0.16 mM). We used 630 nm illumination in order to excite Cy5.5LP dye. Emission was collected at two wavelengths: at 700 nm to observe emission of the FRET donor Cy5.5LP, and at 820 nm to observe emission of the FRET acceptor Cy7.5LP (Fig. 3). In order to evaluate directly FRET changes independently of concentration, we also generated ratio images where emission of the acceptor was divided by the emission of the donor (A/D, Fig. 3). Before injection, only low fluorescence was observed for 700 nm channel and negligible signal at 820 nm (Fig. 3A). Right after injection (15 min), the emission of Cy7.5LP corresponding to FRET increased significantly, while emission intensities collected in the Cy5.5LP channel remained very close to auto-fluorescence levels observed before injection (Fig. 3B). This strong FRET fluorescence suggested that the integrity of our FRET NCs was well preserved *in vivo* 15 min after their parenteral injection. Even more importantly, we obtained high imaging contrast in the FRET channel, where blood circulation could be distinguished, notably in the tail and in the back legs vasculature (see arrows 1 and 2 in Fig. 3B). Strikingly, efficient FRET (high A/D ratio in red) highlighted strongly vascularized organs, particularly liver. Other organs sitting above the liver, such as the lungs and potentially thyroid glands, were also resolved with our FRET NCs. Thus, the use of ratiometric imaging of whole living mice with our FRET NCs provided remarkably high resolution, where both the blood circulation and several important organs were easily revealed.

**Fig. 4:**
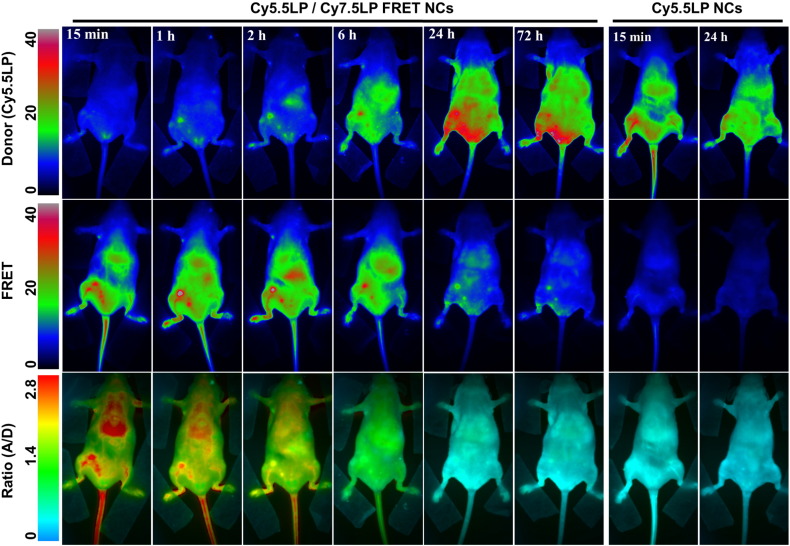
FRET imaging of healthy nude mice at different times after injection with NIR-FRET lipid NCs (1% of Cy5.5LP and Cy7.5LP each) and control NCs containing only Cy5.5LP dye (1%). Upper panels present intensity images of the Cy5.5LP channel (700 nm), middle panels present images of Cy7.5LP channel (820 nm), while the lower panels present ratiometric images (acceptor/donor). The excitation wavelength was 630 nm. Experiment was repeated on three mice, the set of data for the other two mice (15 min post-injection) is reported in the Supplementary information section (Fig. S6).

Further time-course imaging experiment revealed that fluorescence intensities of our NIR dyes and the associated ratio underwent dramatic evolution. The fluorescence intensity gradually increased in the Cy5.5LP channel, and became significant after 6 h, reaching maximal values only after 24 h. On the contrary, overall FRET intensity gradually decreased, so that after 24 h it was much lower than that at Cy5.5LP channel (Fig. 4). The ratio (A/D) imaging confirmed the continuous decay of the FRET signal as the initial red pseudo-color changed to green, and reached the lowest levels (blue) 24 h post-injection (Fig. 4 and S5). Remarkably, this color switch corresponded to ~ 30-fold decrease in the A/D ratio within 24 h, indicating profound changes of FRET in our NCs as well as high sensitivity and dynamic range of the ratio imaging. This experiment reveals, using FRET imaging, the time-course of disintegration of our NCs. When the control NCs, containing only Cy5.5LP were injected, the emission at the donor (Cy5.5LP) channel was much higher than that at the acceptor one, whatever the post-injection time (Fig. 4 and S7). The obtained ratio images gave very similar blue pseudo-coloring all over the mice body for different post-injection times (Fig. S7), in agreement with total absence of FRET in the control NCs. They matched well with the images obtained at 24 h post-injection of the FRET NCs, where the FRET signal was lost (Fig. 4). Interestingly, we obtained high-quality contrast in the Cy5.5LP channel 15 min after injection of our control donor dye-loaded NCs, and could easily discern both the vasculature network as well as highly-vascularized organs such as the liver. However, in contrast to the ratio imaging with FRET NCs, fewer details were observed (*i.e.* no lungs and less blood vessels). The time-lapse experiment showed a gradual decay of the fluorescence intensity from the tail vasculature (tail vein) with complete disappearance of the signal after 24 h (Fig. 4 and S8).

**Fig. 5:**
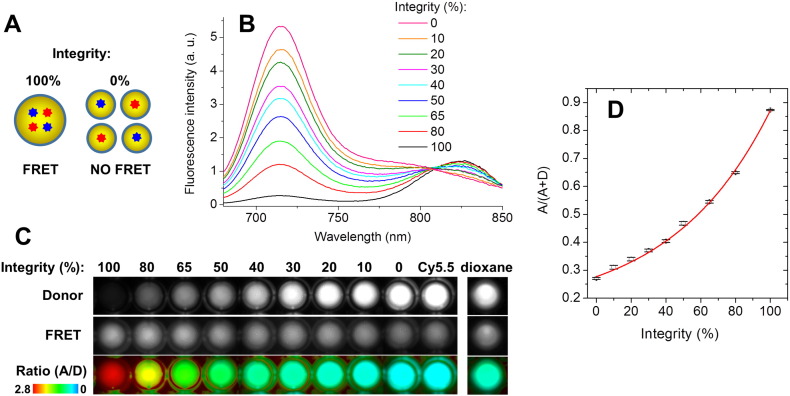
Calibration of ratiometric *in vivo* images using mixtures of intact FRET NCs with NCs containing separately donor and acceptor at low concentration. The mixing is done to preserve the same concentration of dyes: 100% integrity corresponds to FRET NCs (1% of Cy5.5LP and 1% of Cy7.5LP) diluted in PBS 1000-fold from the original formulation, while 0% integrity corresponds to a mixture of NCs containing separately donor (0.1% of Cy5.5LP) and acceptor (0.1% Cy7.5LP), both diluted at 100-fold in PBS. The latter mixture models the disintegrated NCs, where donor and acceptor separate and get diluted in the tissue (A). (B) Fluorescence spectra of the obtained solutions. (C) Fluorescence images of these mixtures acquired with the *in vivo* imaging set-up: donor (Cy5.5LP) channel (upper panels), acceptor (Cy7.5LP) FRET channel (middle panels), and the acceptor/donor ratio images (lower panels). NCs containing only donor dye (Cy5.5LP) and FRET NCs diluted 1000-fold in dioxane (second model of the complete disintegration) are also shown. (D) Calibration curve of A/(A + D) ratio *vs* the level of integrity of NCs obtained based on data in (C) panel. The error bars represent the standard error of the mean (*n* = 3).

**Fig. 6:**
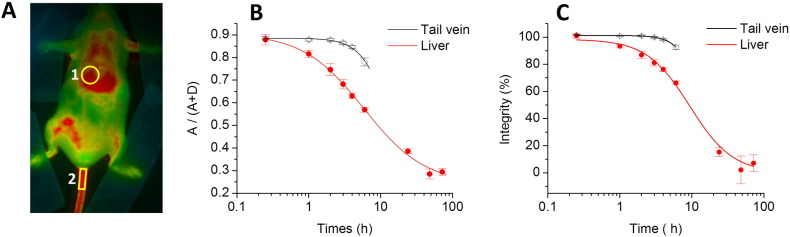
Quantitative analysis of NCs integrity in living mice. (A) Ratio image of healthy mice showing the regions of interest: liver (1) and tail vein (2). (B, C) Analysis of the A/(A + D) ratio (B) and integrity (C) of NCs in different regions of healthy mice as a function of post-administration time. Ratio analysis in the tail vein was done until 6 h, because after that the signal was too low. Three mice were analyzed, the error bars represent the standard error of the mean (n = 3).

To provide connection between observed pseudo-color and the integrity of our NCs, we performed a calibration of our imaging set-up with diluted solutions of NCs containing mixtures modeling different integrity levels. As our dyes did not show significant leakage in serum even within 24 h, we consider that disintegration of NCs observed *in vivo* results in the destruction of the droplets themselves. The latter would lead to the dilution of the liberated dyes in the surrounding tissue especially in their lipid structures (lipid membranes and droplets), producing the loss of FRET. In this case, to model fully disintegrated NCs, we mixed NCs containing separately donor and acceptor dyes at low concentration (0.1%). The final concentration of each dye in this model mixture was identical to that in our FRET NCs, but in this case donor and acceptors dyes were isolated from each other and diluted in the oil of NCs. This system should mimic disintegrated NCs, where liberated donors/acceptor dyes are highly diluted in lipid environments of the tissue. We also prepared mixtures of FRET NCs and NCs containing separated FRET partners, where % of integrity was defined as the fraction of the dyes in the intact FRET NCs with respect to total amount of dyes in the mixture. Decrease in the integrity level produced drastic variation of the two-band emission of NCs with rapid increase in the donor emission and less pronounced decrease in the acceptor emission (Fig. 5B). These changes were expected as the fraction of non-FRET NCs increased. Then, using our *in vivo* imaging setup, we observed that a decrease in the NCs integrity correlated with a rapid increase in the donor channel, with only minor decrease in the acceptor one (Fig. 5C), in line with the spectroscopic data. Ratiometric images of the intact FRET NCs appeared in red, which reliably matched the blood circulation of healthy mice at 15 min after injection (Fig. 4). Subsequently, the decrease in the NCs integrity level produced gradual change of the pseudo-color to yellow, green and then to blue, reflecting the drop of the A/D ratio (Fig. 5C). Remarkably, changes in the pseudo-color of our calibrating solution followed the same trend as those in the ratiometric images of mice in the course of 24 h. The same color switch to blue was observed once the NCs were diluted in dioxane (Fig. 5C), which was an additional model of NCs disintegration. Based on the calibration results, the ratio images obtained in living mice could directly be correlated to NCs integrity *in vivo*. To realize quantitative analysis of the changes in the NCs integrity, we first corrected the data from the background signal in liver and tail vein for both 700 and 820 nm channels, and used them to obtain the A/(A + D) ratio, which is used here as a semi-quantitative measure of the overall FRET efficiency in our samples. This ratio remained stable for 2–3 h post-injection in the tail vein (Fig. 6B), indicating remarkable stability of NCs in the blood circulation. In sharp contrast, this ratio showed significant drop in the liver, indicating significant loss of FRET and thus NCs integrity on the time scale of 2–3 h. Although we cannot provide absolute values of FRET efficiency simply based on the A/(A + D) ratio [48], [53], [54], we could directly correlate this ratio with the integrity level of NCs (Fig. 5D) using calibration images in Fig. 5C, recorded by the same imaging setup with identical emission filters. The obtained plot of A/(A + D) ratio *vs* integrity level of NCs was fitted using a non-linear equation and then used as our calibration curve. Consequently, this allowed us to estimate integrity of our NCs over time for liver and tail vein, directly in living mice (Fig. 6C). This analysis revealed dramatic difference in the stability of NCs in blood circulation and liver. At 6 h of post-administration, the integrity of NCs in the liver decreased to 66 ± 2%, whereas in blood circulation NCs remained practically intact with integrity level reaching 93 ± 2% (Fig. 6C) (*p* < 0.0003, n = 3). The integrity half-life of NCs was 8.2 ± 0.4 h in the liver of healthy mice. Unfortunately, this parameter could not be assessed for the blood circulation since NCs were cleared from the blood on the shorter time scale. Indeed, our experiments with NCs containing only donor dye (Cy5.5LP) (Fig. S7) confirmed that the circulation half-time was 3 ± 1 h (Fig. S8). Therefore, after 6 h, most of NCs were cleared from the blood circulation, so the detected signal in the tail region was produced mainly by surrounding tissues. Thus, the lipid NCs remain stable until their clearance from the blood. On the other hand, our data suggest that liver can disintegrate lipid NCs on the time scale of hours.

**Fig. 7:**
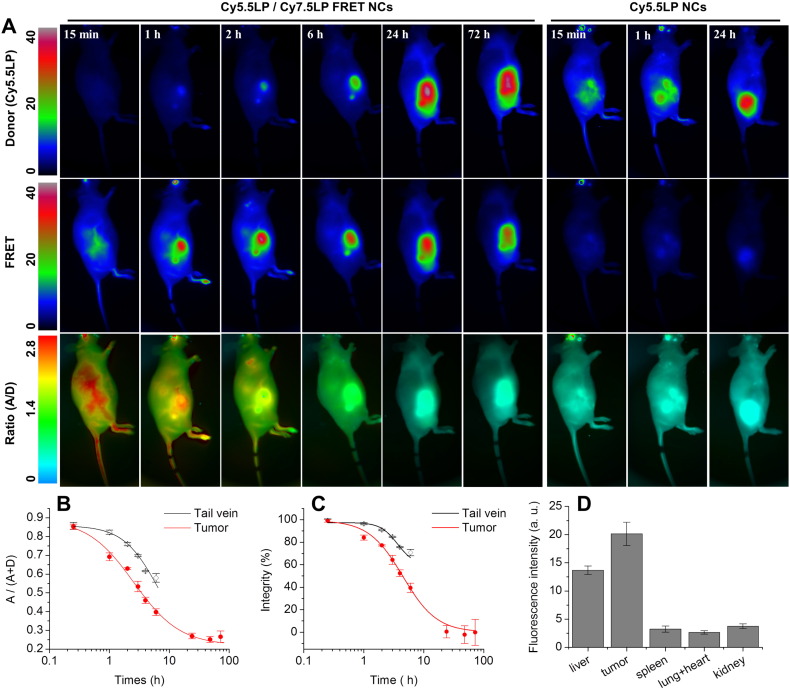
(A) FRET imaging of tumor-bearing nude mice at different times after injected with NIR-FRET lipid NCs (1% of Cy5.5LP and Cy7.5LP each) or control NCs with donor dye only (1% of Cy5.5LP dye). Upper panels present intensity images of the Cy5.5LP channel (700 nm), middle panels present images of Cy7.5LP channel (820 nm), while the lower panels present ratiometric images (acceptor/donor). The excitation wavelength was 630 nm. Experiment was repeated on three mice, the set of data for the other two mice is reported in the Supplementary information section (Fig. S10). (B, C) Analysis of the A/(A + D) ratio (B) and integrity (C) of NCs in different regions of tumor-bearing mice as a function of post-administration time. Three mice were analyzed, the error bars represent the standard error of the mean (n = 3). (D) Fluorescence intensity of organs dissected from animals at 24 h post-administration with NCs containing 1% of Cy5.5LP dye. The error bars corresponds to standard error of the mean (n = 4). Images of organs are presented in Fig. S12.

Next, because NCs bear great potential in tumor targeting, we administrated our FRET NCs to tumor-bearing animals, where murine tumor cells (D2A1) were subcutaneously xenografted in the flank of nude mice 20 days before NCs injection (see photo of one tumor bearing mice in Fig. S4). Thus, assuming that EPR would lead to accumulation of NCs in tumors, the basic question we asked was whether the NCs enter tumor in their intact or disintegrated form. Upon administration of NCs, we observed a significant targeting of the microenvironment of the tumor by our NCs, which led to fluorescence around tumor in the FRET channel and almost negligible fluorescence in the Cy5.5LP channel (Fig. 7A and Fig. S9). This fluorescence signal corresponded to high A/D ratio (observed in red), indicating efficient FRET, similar to that in the tail vessel. Thus, the fluorescence observed in the peri-tumoral region can be assigned to the tumor-associated vasculature, which contains high concentration of intact FRET NCs. Already at 1 h post-injection, the tumor region displayed very strong FRET signal, even though signal collected in the Cy5.5LP channel remained very low (Fig. 7A). In this case, the absolute intensity of the donor channel was only 5.7 ± 1.3 (arbitrary units, a. u.), whereas for control NCs with only donor Cy5.5LP, the intensity in the tumor region at 1 h injection was 26 ± 2 (a. u.). This much lower intensity of the donor channel suggested the efficient FRET-based quenching of the donor inside the FRET NCs accumulated in the tumor. Ratio image at 1 h showed mainly yellow-red color in the tumor region, which indicated that after accumulation in the tumor the FRET remained very high despite a small decrease. These observations suggested that (*i*) the lipid NCs underwent a specific, rapid and efficient accumulation into the xenografted tumor, through the tumor-associated vasculature, and (*ii*) they remain, to a large extent, intact for at least 1 h within tumor region. Intensity of the FRET signal in tumor reached maximum values 2 h post-injection and then remained stable over the time-course of the experiment. In contrast, the fluorescence intensity in the donor channel increased slowly over the first 6 h post-injection, but drastically increased 24 h post-injection. The ratiometric imaging showed that relative intensity of the FRET acceptor decreased continuously with time, reaching the lowest values after 24 h of incubation. Experiments with control NCs containing only donor dye Cy5.5LP confirmed rapid accumulation of NCs into the tumor region already at 1 h post-injection (Fig. 7A). The acceptor channel remained dim in the tumor region, in line with expected absence of FRET. The intensity in the donor channel continued to gradually increase over time, reaching saturation after 24 h, whereas in the acceptor channel the intensity remained poor (Fig. S11). Imaging of mice organs (Fig. S12) dissected from animals at 24 h post-administration revealed that fluorescence of tumor was slightly brighter than that of liver (by 30%, *p* = 0.02, n = 4). By contrast, fluorescence of other studied organs (spleen, lungs, heart and kidneys) was many-fold lower (*p* ≤ 0.0002, n = 4), suggesting that both tumors and liver are the main targets for accumulation of our NCs, in line with the whole animal *in vivo* imaging data.

After accounting for the background fluorescence, we quantified the FRET proximity ratio in tumor-bearing mice by analyzing the A/(A + D) ratio (Fig. 7B) and then converted it to the NCs integrity level for different post-administration times (Fig. 7C). Importantly, after 2 h, when the signal of NCs from the tumor region was already very strong (Fig. 7A), the integrity of NCs was still at 77 ± 1% (Fig. 7C), indicating that they could enter the tumor with minimal loss of integrity. Nevertheless, the loss of integrity was faster than in the blood circulation. Indeed, in tail vein after 6 h NCs integrity was at 71 ± 3%, whereas for tumor it dropped already to 40 ± 4% (Fig. 7C) (*p* < 0.003, n = 3). The integrity half-life for our NCs in tumor was 4.4 ± 0.3 h, which was even faster than in the liver of healthy mice (8.2 ± 0.4 h, *p* < 0.001, n = 3). We provide the first evidence for the kinetics of disintegration of lipid NCs in tumor bearing mice *in vivo* and show that the lipid NCs preserve their content in the blood circulation, then they can enter tumors with minimal loss of integrity and finally after accumulation in the tumors they disintegrate on the time scale of hours. One should also note that disintegration of NCs inside blood circulation of tumor bearing mice was considerably faster than in healthy mice, which can be seen from the integrity levels at 6 h of post-administration (71 ± 3% *vs* 93 ± 2%, *p* < 0.003, n = 3). On the other hand, the clearance from blood circulation, measured using control NCs bearing only Cy5.5LP (Fig. S11) in tumor-bearing mice was similar to that in healthy mice (Fig. S8).

In conclusion, we propose here an original approach to obtain near-infrared lipid nanocarriers with efficient FRET and excellent brightness. It is based on hydrophobic counterions that increase many-fold the solubility of cyanines dyes in oil and thereby enable their high loading inside NCs, required for FRET. We provide here clear demonstration that these new NCs allow reliable imaging and monitoring NCs' integrity *in vivo*. Notably, we provide here whole-animal imaging with apparently higher resolution compared to existing FRET systems for *in vivo* imaging [9], [24], [25]. Importantly, we were able to accurately resolve the follow-up of NCs integrity directly in the blood circulation of living mice, which was possible before only by using dedicated intravital microscopy [9], [27]. The high-quality of the obtained images is probably due to relatively high fluorescence quantum yield, dye-loading and injection dose, as well as optimal NIR spectral excitation and emission windows of our FRET NCs. Moreover, by dividing two emission channels, we generated high quality ratiometric images capable of resolving some features of the vascular system and tissues that are usually not assessable by live whole-animal fluorescence imaging. The intensity ratio is the absolute parameter, which is almost independent on the concentration of the imaging agent and excitation light intensity [31], [32], [55], [56]. Therefore, the ratio signal provides quantitative information about the changes in the emission spectra of the probe in the imaged system. In our case, due to calibration of the spectral response of the FRET NCs directly under the *in vivo* imaging setup, we achieved a reliable read-out of the integrity of our NCs by ratiometric analysis, which would not be possible using absolute intensity measurements. It should be noted that in addition to calibration of the imaging system, this method requires subtraction of the background, which is relatively high in small animal fluorescence imaging. One important point that is difficult to take into account is the difference in the penetration depth for 700- and 820-nm light [30], which may affect the precision of the quantitative analysis.

The described methodology enabled, for the first time, to quantify by *in vivo* fluorescence imaging the integrity of NCs in different regions of healthy and tumor-bearing mice. It revealed that injected NCs remain nearly intact after 6 h in the blood circulation of healthy mice, whereas their integrity significantly dropped to 66 ± 2% in the liver of healthy mice, and up to 40 ± 4% in tumors. Nevertheless, lipid NCs appeared remarkably stable as they could enter tumors rapidly with minimal loss of integrity, showing for instance 77 ± 1% of the integrity at 2 h of post-injection. Thus, NCs can preserve their content in the blood circulation, enter tumors in nearly intact form and then release this content on the time scale of hours. This data shows the strong potential of NCs as efficient carriers of drugs and contrast agents. The remarkable stability of lipid NCs is an important finding of this work. Commonly, nanoemulsions are considered as liquid systems that should not be stable enough *in vivo* and require, for instance, specially designed surface of lipids with long PEG chains [25], [36]. However, in the present work, we showed for the first time that nanoemulsion droplets built only from oil (medium chain triglycerides) and a surfactant (Cremophor® ELP) exhibited no signs of disintegration in blood circulation for hours. This result contrasts strongly with the poor capacity of these NCs to keep medium polar molecules like Nile Red, which is rapidly released in biological media [41], [57].

Our NCs showed remarkably efficient accumulation in tumors, which can be clearly assigned to the EPR effect originating from the leaky nature of the tumor vesicles [15], [58]. The efficiency of the passive EPR tumor accumulation is generally proportional to the circulation time of the agent in the bloodstream. In this respect, our NCs present dense PEG shell which is known to prolong the circulation time of nanoparticles [59].

Finally, the key finding that has never been shown for lipid NCs is their capacity to accumulate in tumors in nearly intact form. This property is known for polymer nanoparticles, which are established drug delivery vehicles into tumors [60], [61], and inorganic nanoparticles, such as quantum dots covered with robust organic shell [9], [62]. The capacity of our nano-emulsion droplets to preserve their integrity after accumulation in tumors is particularly remarkable given the liquid nature of their core. This result shows the strong potential of these lipid NCs as nanoscale platform for *in vivo* imaging, drug delivery and image guided surgery.

## Appendix A Supplementary data

Supplementary material. Image 2
