# Supplementary material for: Integrity of lipid nanocarriers in bloodstream and tumor quantified by near-infrared ratiometric FRET imaging in living mice
Source: J Control Release. 2016 Aug 28;236:57–67. doi: 10.1016/j.jconrel.2016.06.027 (PMC4968657; doi:10.1016/j.jconrel.2016.06.027)
Supplement: Supplementary file 1 — Supplementary material. [file mmc1.pdf]

Supporting information

Integrity of lipid nanocarriers in bloodstream and tumor quantified by near-infrared ratiometric FRET imaging in living mice

Redouane Bouchaala, Luc Mercier, Bohdan Andreiuk, Yves Mély, Thierry Vandamme, Nicolas Anton, Jacky G. Goetz, Andrey S. Klymchenko

Synthesis

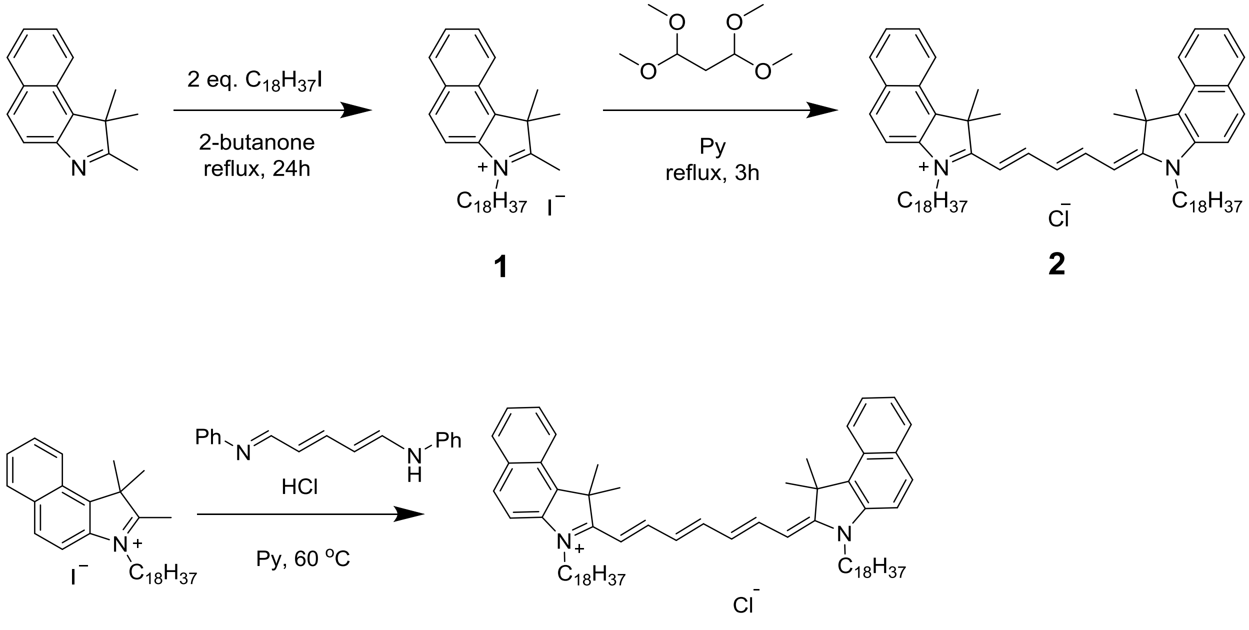

Scheme 1. Synthesis of dioctadecylcyanines 5.5 (**2**) and 7.5 (**3**).

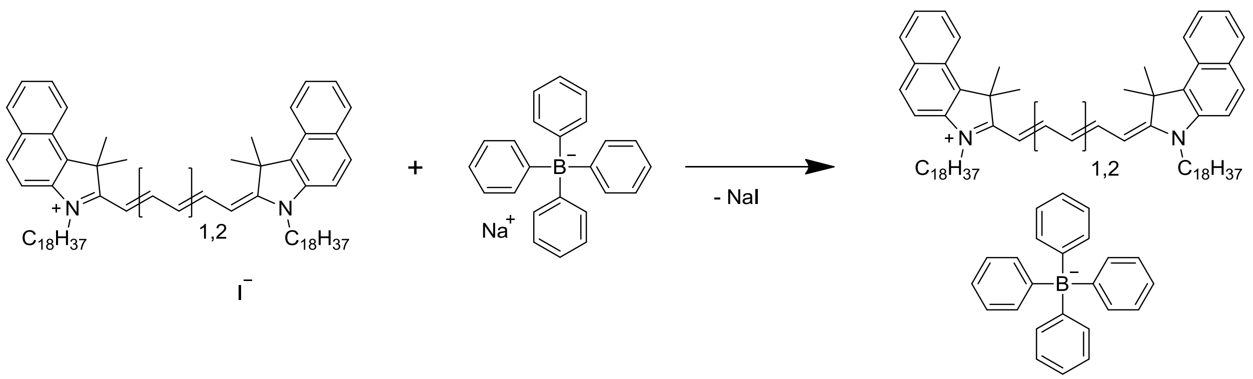

Scheme 2. Synthesis of dioctadecylcyanines 5.5 and 7.5 salts with tetraphenylborate counterion: Cy5.5LP and Cy7.5LP, respectively).

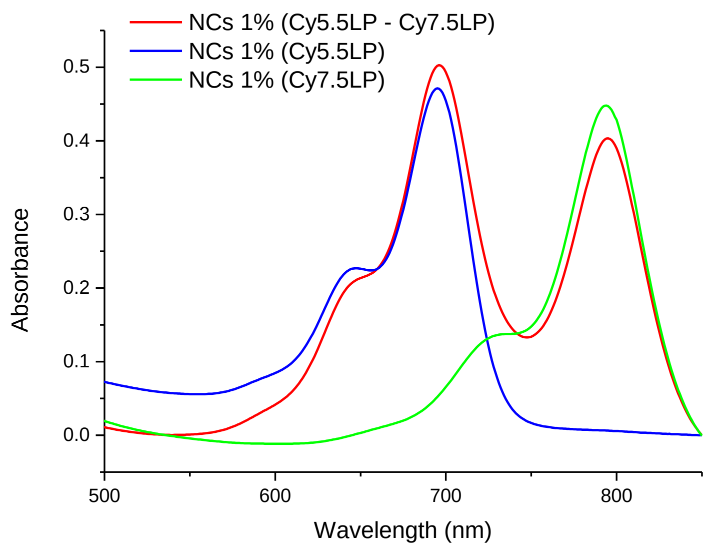

Figure S1. Absorption spectra of NCs encapsulating Cy5.5LP, Cy7.5LP and their mixture. NCs were diluted 500-fold from in water from the original formulation.

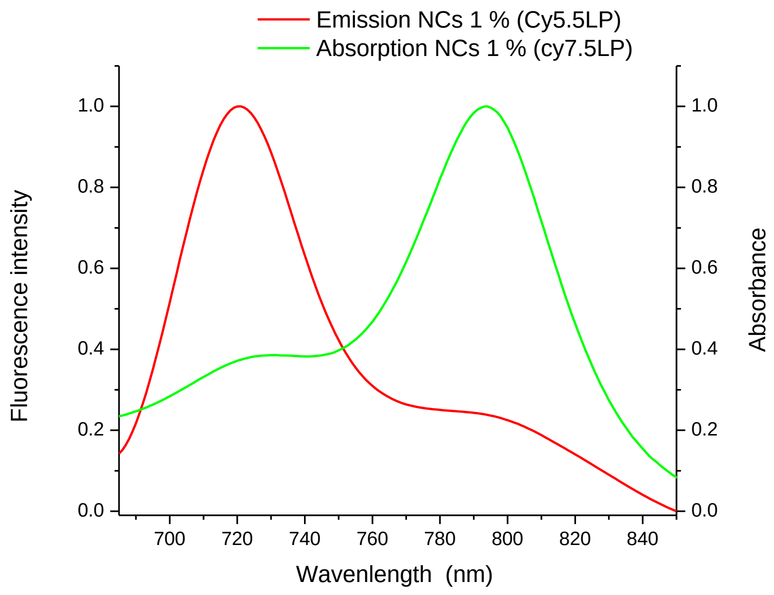

Figure S2. Normalized fluorescence spectrum of NC encapsulating 1% of Cy5.5LP and the absorption spectrum of NC encapsulating 1% of Cy7.5LP showing good spectral overlap, required for FRET. NCs were diluted in water 500-fold from the original formulation.

Table S1. Hydrodynamic diameter and polydispersity index obtained by DLS and the fluorescence quantum yield of lipid nanocarriers encapsulating different NIR dyes.[a]

	Size (nm)	PDI	QY (%)
NC, 1% Cy5.5LP	94	0.165	27
NC, 1% Cy5.5LP / Cy7.5LP	91	0.191	11
NC, 1% Cy7.5LP	90	0.108	5

[a] Statistics by volume was used in the size analysis. PDI is a polydispersity index. QY is a fluorescence quantum yield, measured using DiD dye in methanol (QY = 33 %) as reference [1].

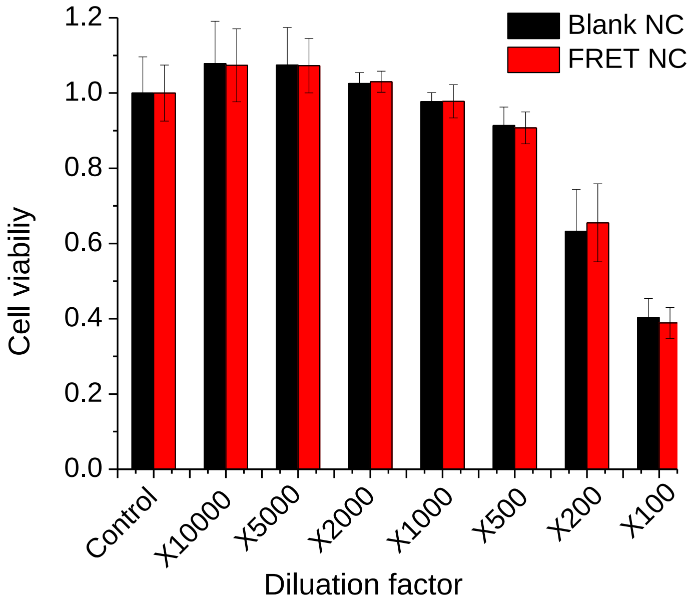

Fig. S3. Cytotoxicity of lipid nanocarriers loaded with FRET pair (Cy5.5LP and Cy7.5LP, 1% each). Cell viability was measured by MTT assay on HeLa cells incubated for 24 h with NCs without and with Cy5.5LP and Cy7.5LP (1% each) at different dilutions from the original formulation. The error bars correspond to standard error of the mean (n = 6).

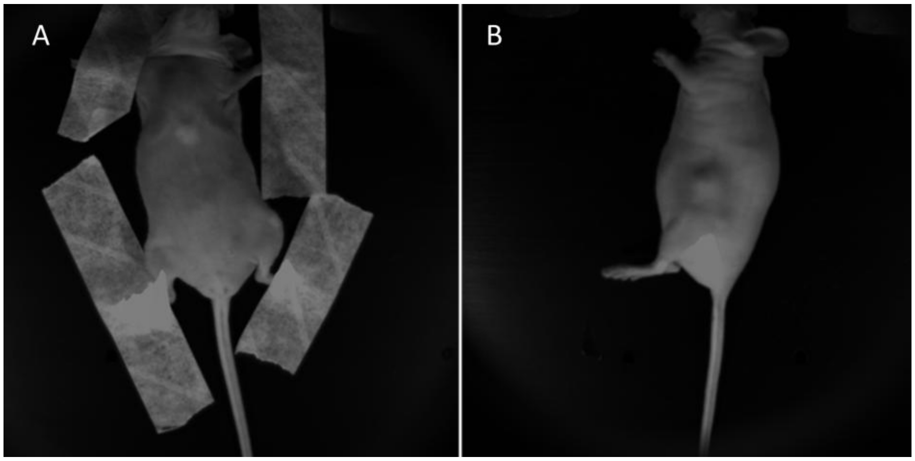

Figure S4. Photos of healthy (A) and tumor-bearing (B) mice which were used in the FRET imaging shown in Figures 3, 4, S3 and S5.

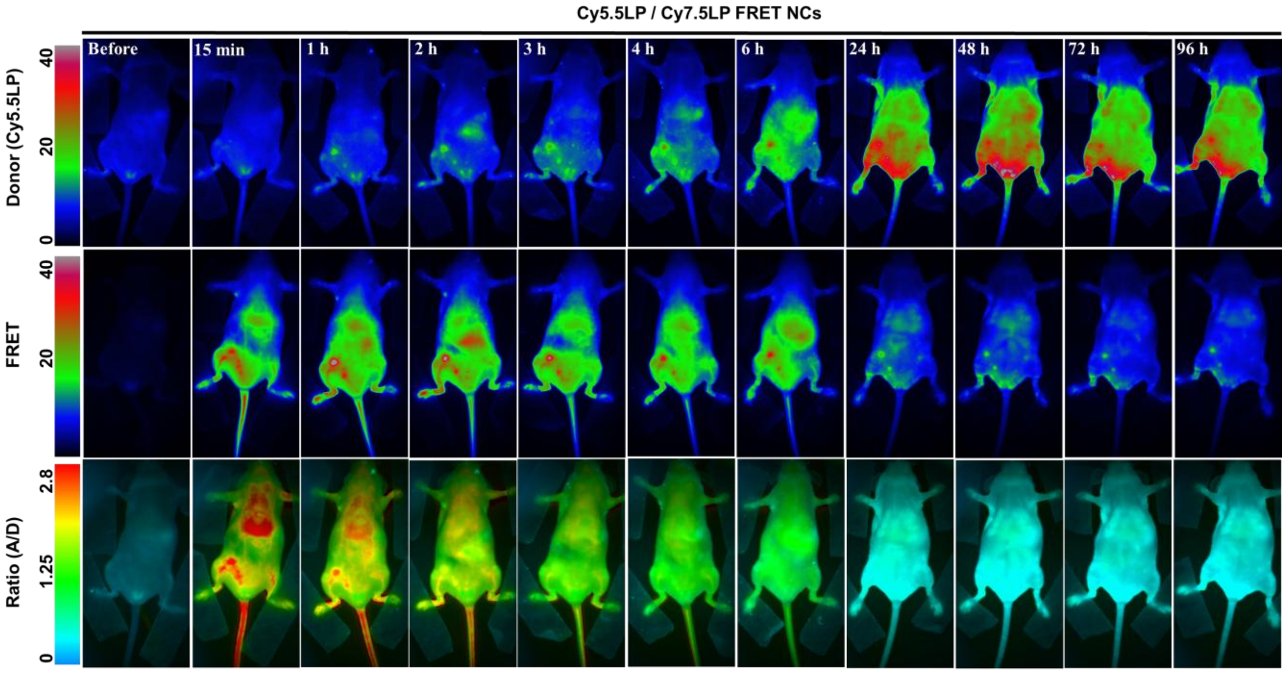

Figure S5. FRET imaging of healthy mice at different times after injection with NIR-FRET lipid nanocarriers. NCs contained 1% of donor Cy5.5LP and acceptor Cy7.5LP. Upper panels present intensity images of the Cy5.5LP channel (700 nm), middle panels present images of Cy7.5LP channel (840 nm), while the lower panels present ratiometric images (acceptor / donor).

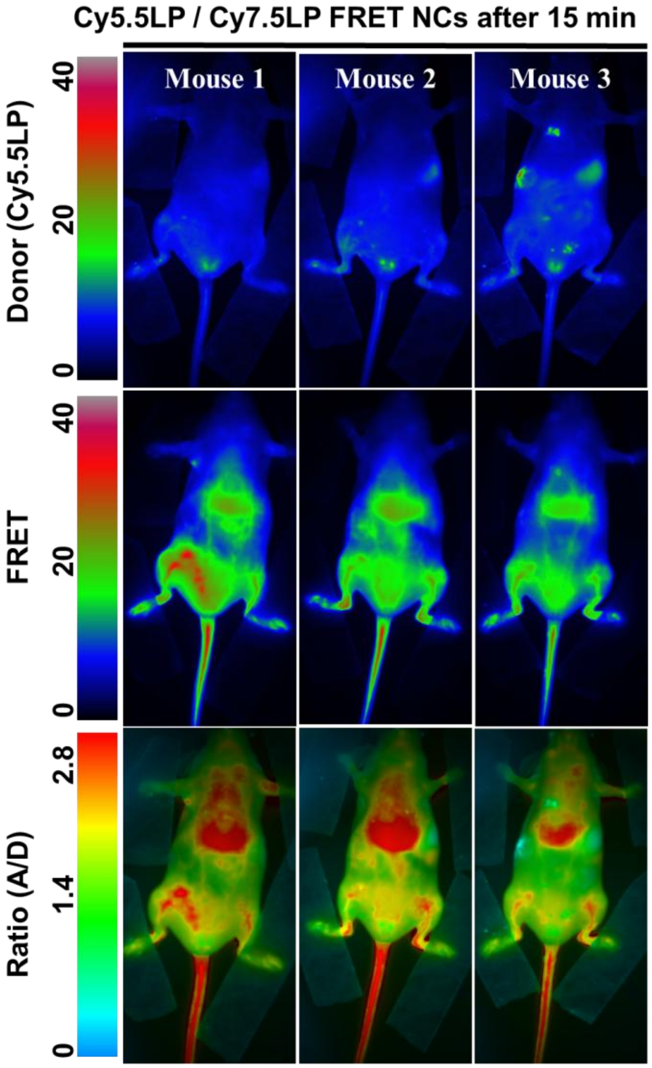

Figure S6. FRET imaging of three different healthy mice at 15 min after injection with NIR-FRET lipid nanocarriers. NCs contained 1% of donor Cy5.5LP and acceptor Cy7.5LP. Upper panels present intensity images of the Cy5.5LP channel (700 nm), middle panels present images of Cy7.5LP channel (840 nm), while the lower panels present ratiometric images (acceptor / donor).

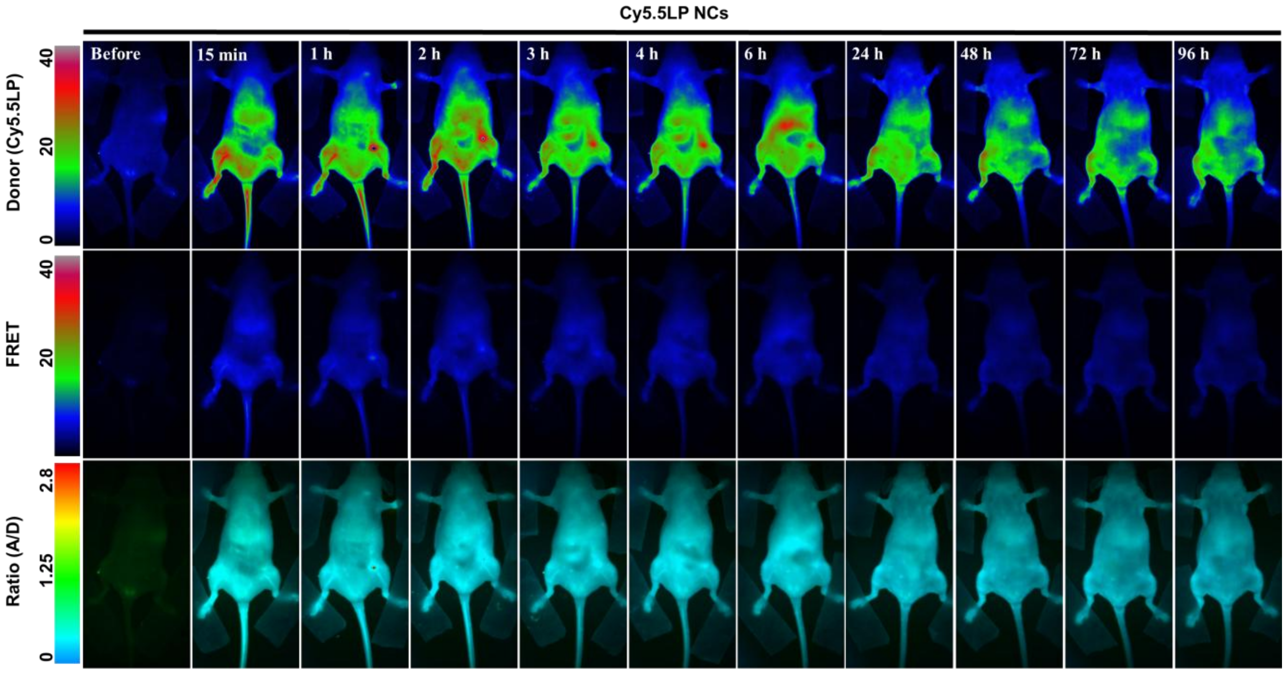

Figure S7. FRET imaging of healthy mice at different times after injected control lipid nanocarriers encapsulating only 1% of Cy5.5LP dye. Upper panels present intensity images of the Cy5.5LP channel (700 nm), middle panels present images of Cy7.5LP channel (840 nm), while the lower panels present ratiometric images (acceptor / donor).

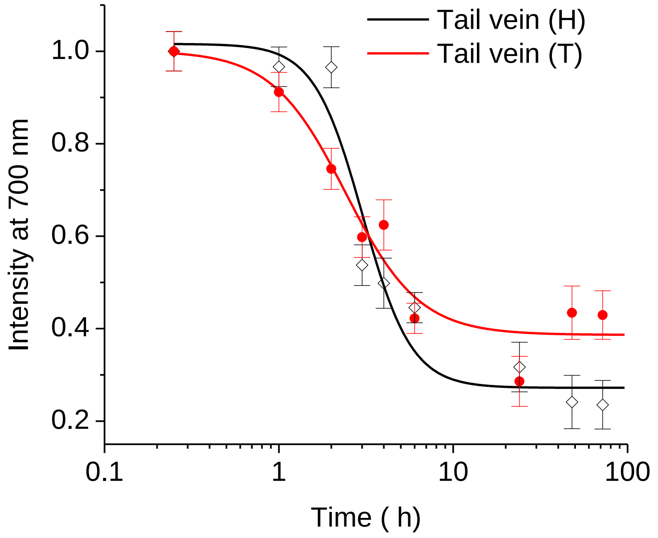

Figure S8. Decay of the fluorescence intensity of the control NCs (Cy5.5LP only) in tail vein of healthy (H) and tumor-bearing (T) mice.

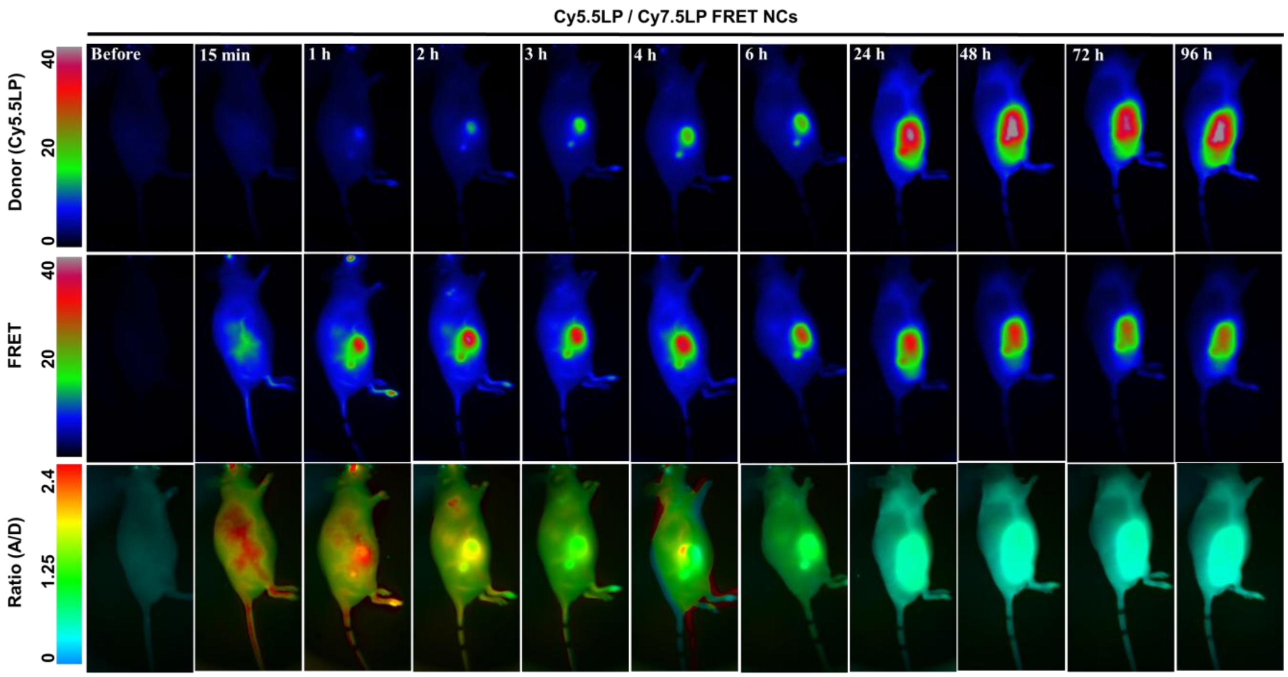

Figure S9. FRET imaging of tumor-bearing mice at different times after injection of FRET lipid nanocarriers. NCs contained 1% of donor Cy5.5LP and acceptor Cy7.5LP. Upper panels present intensity images of the Cy5.5LP channel (700 nm), middle panels present images of Cy7.5LP channel (840 nm), while the lower panels present ratiometric images (acceptor / donor).

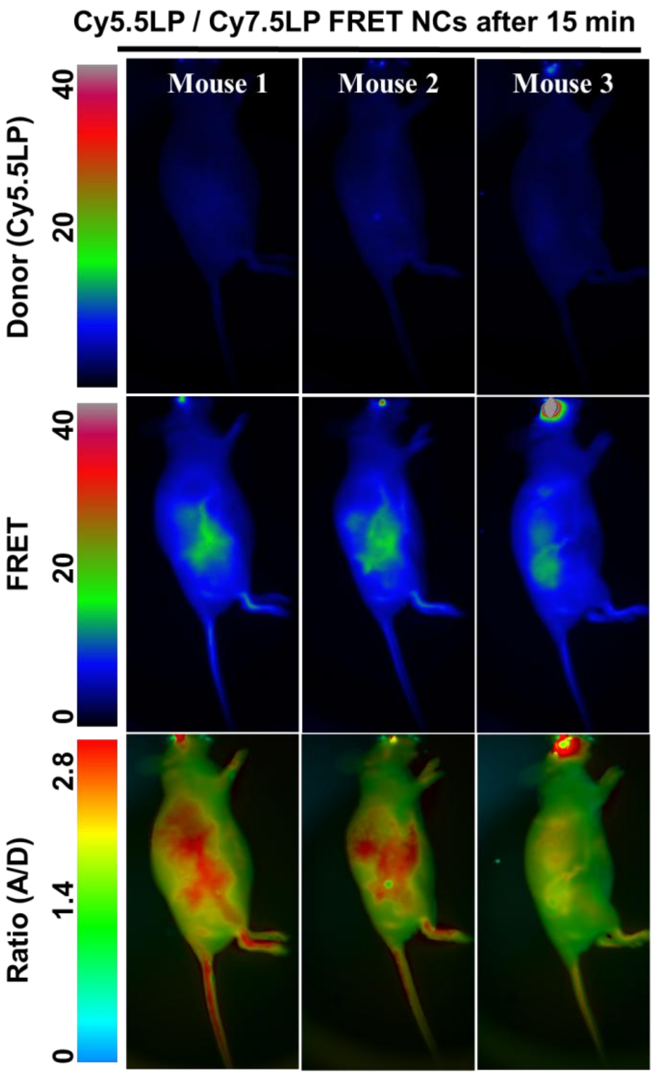

Figure S10. FRET imaging of tumor-bearing mice 15 min after injection with NIR-FRET lipid nanocarriers. NCs contained 1% of donor Cy5.5LP and acceptor Cy7.5LP. Upper panels present intensity images of the Cy5.5LP channel (700 nm), middle panels present images of Cy7.5LP channel (840 nm), while the lower panels present ratiometric images (acceptor / donor).

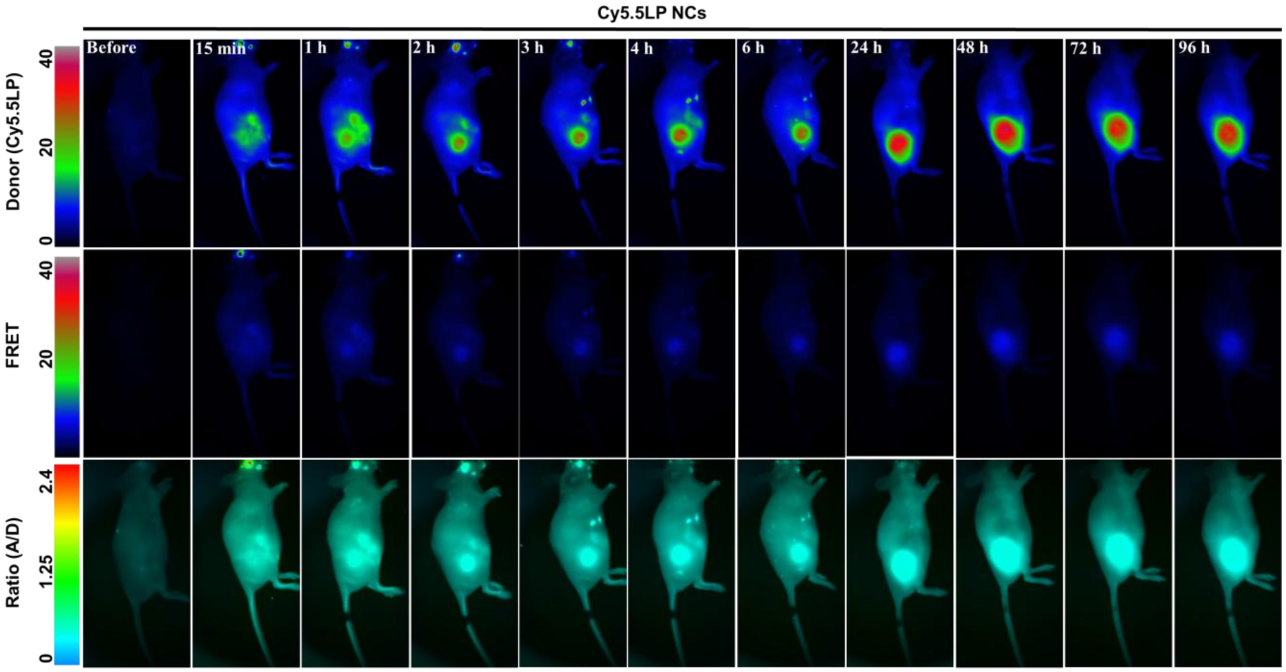

Figure S11. FRET imaging of tumor-bearing nude mice at different times after injected control lipid nanocarriers encapsulating only 1% of Cy5.5LP dye. Upper panels present intensity images of the Cy5.5LP channel (700 nm), middle panels present images of Cy7.5LP channel (840 nm), while the lower panels present ratiometric images (acceptor / donor).

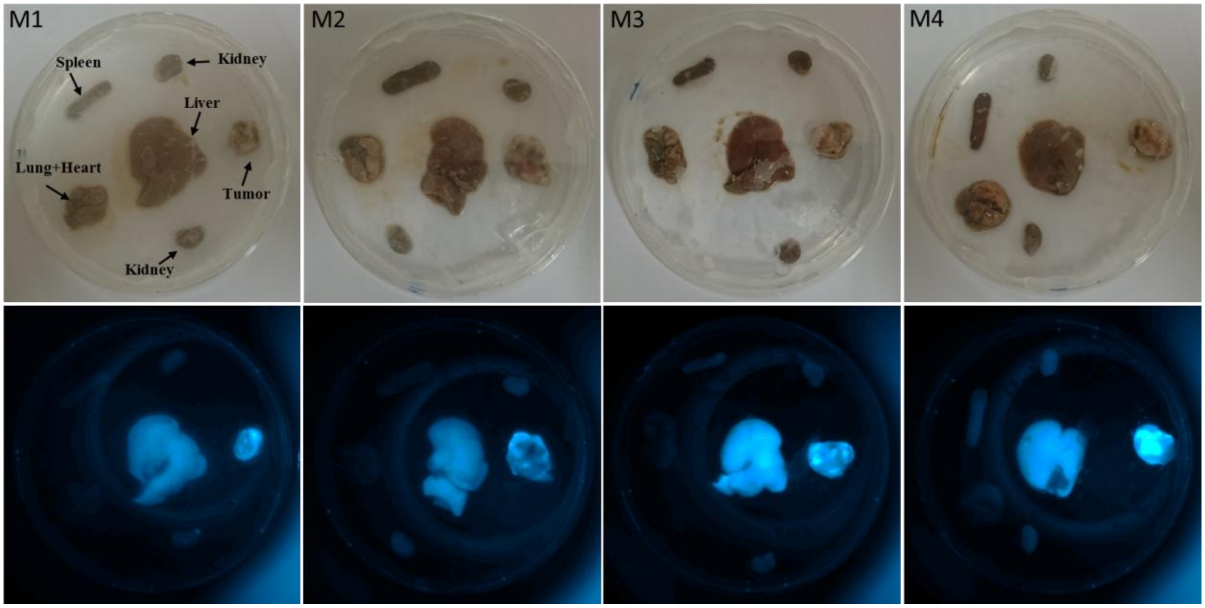

Figure S12. Ambient light photos (A) and NIR fluorescence images of organs of four mice injected with Cy5.5LP NCs 24h earlier. The excitation wavelength was 630 nm, while the emission was detected at 700 nm. Before the injection, NCs solution was diluted 1000-fold in PBS, similarly to all *in vivo* imaging experiments.

References

[1] Texier I, Goutayer M, Da Silva A, Guyon L, Djaker N, Josserand V, et al. Cyanine-loaded lipid nanoparticles for improved in vivo fluorescence imaging. J Biomed. Opt. 2009;14:054005.
